# An Unusual Presentation of Left Ventricular Non-compaction Cardiomyopathy in an Elderly Patient

**DOI:** 10.7759/cureus.15112

**Published:** 2021-05-19

**Authors:** Hesham Afify, Somshukla Ghosh, Melissa Khalil, Mark R Milunski

**Affiliations:** 1 Internal Medicine, University of Central Florida/HCA GME Consortium, Greater Orlando, USA; 2 Cardiology, Orlando Veterans Affairs Medical Center, Orlando, USA

**Keywords:** left ventricular non-compaction cardiomyopathy, non-ischemic cardiomyopathy

## Abstract

Left ventricular non-compaction cardiomyopathy (LVNC) is a rare cardiomyopathy. The true prevalence of LVNC is unclear. The clinical presentation of LVNC varies widely from asymptomatic to end-stage heart failure or sudden cardiac death, and the diagnostic criteria are not standardized. Moreover, there is an increased risk for thromboembolic events with LVNC. We present an unusual case of LVNC first diagnosed in a septuagenarian.

## Introduction

Left ventricular non-compaction cardiomyopathy (LVNC) is a primary genetic cardiomyopathy which results from the arrest of compaction of intertrabecular recesses in the left ventricular myocardium between 12 and 18 weeks of gestation leading to deep trabeculations communicating with the left ventricular cavity. It usually presents with heart failure, arrhythmias, or thromboembolic complications. LVNC is largely diagnosed in the pediatric population. However, it is increasingly recognized later in life in patients with heart failure. Although the occurrence of LVNC is rare, its true prevalence is unknown in the absence of a universally accepted diagnostic criteria. Treatment focuses on symptomatic management of heart failure, anticoagulation, and implantable cardiac defibrillators. Further studies are required for definitive diagnostic criteria and treatment guidelines.

## Case presentation

A 78-year-old Hispanic male with a past medical history of ischemic cardiomyopathy and end-stage renal disease presented to the emergency department (ED) with signs and symptoms of volume overload after missing his hemodialysis session. The patient complained of shortness of breath, paroxysmal nocturnal dyspnea, orthopnea, and dyspnea on exertion for the last three days before admission. He had a known history of true apical aneurysm confirmed on computerized tomography angiography. The patient was on chronic anticoagulation regimen for questionable apical thrombus on transthoracic echocardiography (TTE). Upon initial presentation to the ED, the patient complained of worsening shortness of breath, and was found to have fine bibasilar lung crackles, no jugular venous distention, and normal heart sounds on physical examination. Electrocardiogram (ECG) was performed and showed normal sinus rhythm with poor R-wave progression. No significant changes were found on ECG when compared with previous studies. Chest X-ray showed findings suggestive of early pulmonary vascular congestion. Initial troponin was mildly elevated at 0.046 ng/mL which subsequently trended down after an urgent hemodialysis session along with improvement in the patient’s breathing status.

**Figure 1:**
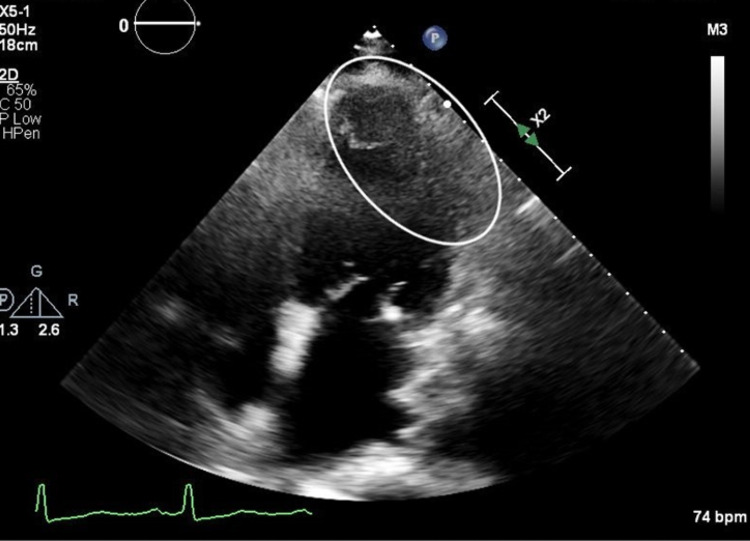
Transthoracic echocardiogram showing a 2.2 × 2.3 cm globular structure in left ventricular apex with an abnormal appearance of the apical lateral segment.

A TTE was obtained which demonstrated a 2.2 × 2.3 cm globular structure in left ventricular apex with an abnormal appearance of the apical lateral segment (Figure 1).

**Figure 2:**
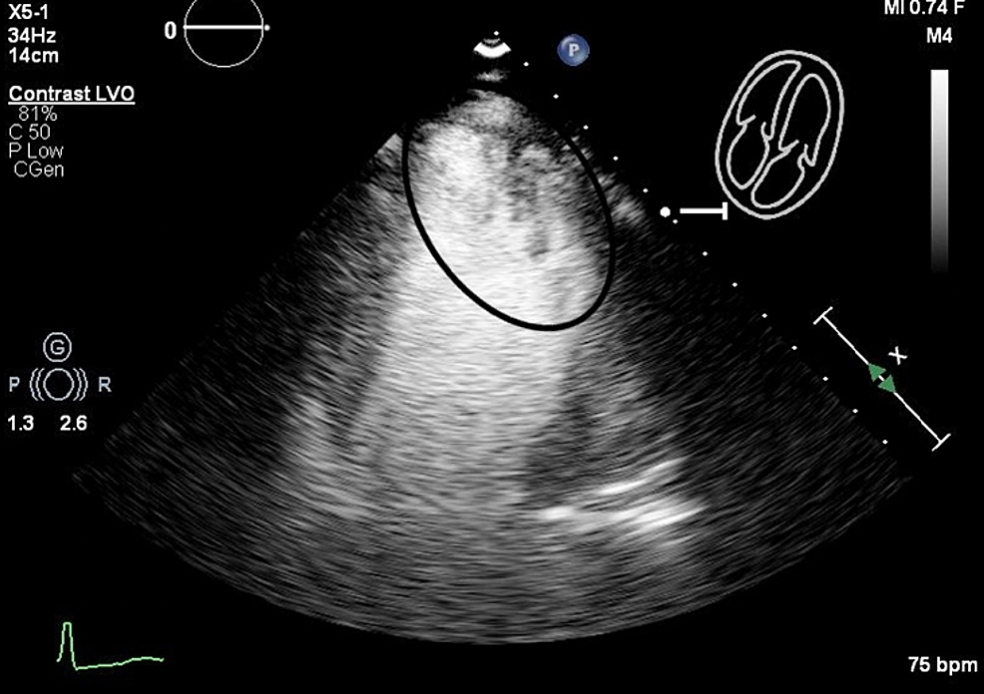
Perflutren-enhanced images demonstrating heavy trabeculation in the left ventricular apex and apical lateral segment.

Doppler pattern was suggestive of either canalization of the apical mass or hypertrabeculation presenting as an unusual variation of LVNC with focal involvement of the left ventricular myocardium. Perflutren-enhanced images demonstrated heavy trabeculation in the left ventricular apex and apical lateral segment (Figure 2).

Cardiac magnetic resonance imaging confirmed the diagnosis of LVNC. Anticoagulation was continued by the team to reduce the thromboembolic risk associated with LVNC.

## Discussion

LVNC is identified as a primary genetic cardiomyopathy which results from the arrest of compaction of intertrabecular recesses in the left ventricular myocardium between 12 and 18 weeks of gestation [1]. This results in the presence of deep trabeculations communicating with the left ventricular cavity. Multiple genetic variants play a role in the development of LVNC and contribute to the classification of LVNC as a primary genetic cardiomyopathy by the American Heart Association in 2006 [2]. However, left ventricular non-compaction has been shown to occur in patients with chronically increased left ventricle pressure [3]. Gati et al. have suggested that LVNC may be over-diagnosed and question whether the myocardial morphology seen with LVNC is truly LVNC or an epiphenomenon associated with increased preload [3]. LVNC has no specific symptoms per se, and it can present with symptoms of heart failure, stroke, or arrhythmias [4]. Thromboembolic complications can occur due to the stagnation of blood flow within the trabeculae of the non-compacted muscle [5].

In isolated LVNC, genetic defects were detected in 35-40% of patients with *MYH7* as the most involved gene. Multiple genes are implicated in the development of LVNC, including coding genes for sarcomeres, cytoskeletal, or ion channel proteins. In a study by Klaassen et al., nine distinct mutations in genes encoding β-myosin heavy chain, α-cardiac actin, and cardiac troponin T were associated with LVNC [6]. In a study by Li et al. on a Chinese population, 38% of LVNC patients were found to carry a pathological genetic variant, with 69% being sarcomere genetic variants as opposed to 31% non-sarcomere variants. The most involved genes were *TTN*, *MYH7*, *MYBPC3*, and *DCP* in Chinese patients [7]. The presence of these genetic variants was associated with a higher risk of death or need for cardiac transplantation in comparison with the genetic negative group [7]. LVNC can occur in isolation or in combination with other congenital heart diseases. The mutations observed in LVNC are shared with other cardiomyopathies [6]. Other congenital conditions associated with LVNC include hypertrophic cardiomyopathy, dilated cardiomyopathy, arrhythmogenic right ventricular cardiomyopathy, and endocardial fibroelastosis. LVNC has been observed in athletes and pregnant women with no adverse events or associated genetic abnormalities. In one study, athletes met the criteria for non-compaction with increased left ventricular trabeculation fulfilling criteria for LVNC and had no observed adverse events [8]. Presumed pregnancy-induced new left ventricular trabeculations have been described, which were reversible in 75% of the patients studied throughout a two-year follow-up [9]. Taken together, these findings suggest that increased ventricular preload can increase the trabeculation seen in the left ventricular myocardium.

There is no universally accepted diagnostic criteria for LVNC. As a result, the true prevalence of LVNC can only be estimated and has been the subject of several studies with results differing based on the imaging modality used. Echocardiography, with or without contrast imaging, has been the gold standard for diagnosis; however, the lack of unified echocardiographic criteria for diagnosis has led to the variability in the figures quoted for the true prevalence of this condition. Criteria suggested by Chin et al. identifies LVNC by the presence of prominent left ventricular trabeculations in relation to the compacted myocardium during diastole in the parasternal short-axis and/or apical views [10]. The criteria proposed by Jenni et al. depend on assessing the Doppler blood flow in the endomyocardial recesses at end-systole on the short-axis parasternal view [11]. Using the Jenni echocardiographic criteria in their original form, the estimated prevalence was 2.76% compared with 0.56% when a non-compacted-to-compacted (NC/C) ratio of ≥2 was used. A study by Söllberger et al. defined non-compacted myocardium by the presence of three or more trabeculations [12]. In a meta-analysis using data published in the literature since 1946, the prevalence of LVNC was 1.28% in the echocardiography group and 14.79% in a group studied with cardiac magnetic resonance imaging (CMR). There was a 12-fold increase in the prevalence of LVNC when using CMR [13]. Moreover, CMR with late-gadolinium enhancement (LGE) served as a predictor of adverse outcomes in LVNC patients. LVNC patients with LGE had a worse prognosis compared with no LGE [14].

Understanding LVNC pathophysiology and its genetic predisposition is crucial for management. There is no curative treatment for LVNC, and treatment mainly focuses on addressing the potential complications associated with LVNC. There is no recommendation with respect to genetic testing in patients with isolated LVNC but should be considered in individuals with a co-occurring cardiomyopathy and family history. LVNC can present as heart failure with either reduced or preserved ejection fraction. The prevalence of heart failure associated with LVNC is estimated to be about 3-4% in hospitalized patients [15]. In a meta-analysis on the prognostic significance of LVNC, it was found that the incidence of cardiovascular and all-cause mortality in LVNC is comparable to non-ischemic dilated cardiomyopathy. However, LVNC has a higher heart failure admission rate [16]. Additionally, there is a difference in survival in patients with isolated apical and mid-basal LVNC. Patients with isolated apical LVNC and preserved left ventricular ejection fraction have similar outcomes to the general population [17]. LVNC cardiomyopathy is associated with a higher burden of arrhythmia, and prophylactic placement of automatic implantable cardioverter defibrillators may have a mortality benefit in selected cases [18]. Patients with LVNC are prone to left ventricular thrombus formation with increased risk of stroke [19]. However, the presence of LVNC alone is not sufficient for instituting prophylactic anticoagulation. The use of prophylactic anticoagulation is controversial as the suggestion for its use is extrapolated from case series and not randomized controlled trials. Some authors conclude that the presence of LVNC alone should suffice to start anticoagulation [20]. On the other hand, there is a growing body of literature that recommends starting anticoagulation in the presence of intracardiac thrombus, atrial fibrillation, or history of venous thromboembolism [5]. Finally, cardiac transplantation offers a viable option for definitive treatment after exhausting other management alternatives when LVNC is associated with end-stage heart failure.

## Conclusions

LVNC cardiomyopathy is a congenital and possibly an acquired heart disease with growing prevalence and recognition owing to better diagnostic modalities. However, lack of standardized diagnostic criteria poses the risk of missing LVNC as the spectrum of clinical presentation ranges from asymptomatic to sudden cardiac death. Although CMR has higher sensitivity than echocardiography in detecting LVNC, it can lead to over-diagnosis, and so care needs to be exercised when interpreting the results of these studies. If the prevalence of LVNC is as high as suggested by some studies, then the correct diagnosis of LVNC is important as management and genetic screening is distinct from other cardiomyopathies. Further studies are required to define the diagnostic criteria, the implications of genetic testing, and the true risk of thromboembolism in patients with LVNC.
